# Digital Ischemia as an Unusual Manifestation of Hodgkin's Lymphoma

**DOI:** 10.1155/2018/1980749

**Published:** 2018-08-19

**Authors:** Fiorella Villano, Adriana Peixoto, Eloísa Riva, Carina Di Matteo, Lilián Díaz

**Affiliations:** ^1^Department of Hematology, Médica Uruguaya (MUCAM), Montevideo, Uruguay; ^2^Laboratory of Pathology Carina Di Matteo, Montevideo, Uruguay

## Abstract

Digital ischemia is associated with atherosclerotic, thromboembolic, or connective tissue diseases. Less often, it can be related to malignancy. Paraneoplastic vascular acrosyndromes (Raynaud's syndrome, acrocianosis, and acronecrosis) are associated with adenocarcinoma and less frequently with hematological malignancies. We report the case of a 45-year-old male, smoker, with a 10-day history of pain, cyanosis, and progressive digital necrosis in both hands. In the previous four months, he noticed painless mass in the right axillary gap, drenching night sweats, and weight loss. Physical examination at admission highlighted necrotic lesions on the distal phalanges of both hands (except the thumbs), enlarged lymph nodes in right axillary, and right supraclavicular gaps. Arteriography of upper limbs demonstrated a distal stop in all bilateral digital arteries. Digital ischemia was interpreted as a paraneoplastic phenomenon after other common etiologies were ruled out. Amputation of three phalanges was required due to necrosis. Biopsy of axillary nodes demonstrated nodular sclerosis classical Hodgkin's lymphoma (HL). The patient started conventional ABVD protocol (doxorubicin, bleomycin, vinblastine, and dacarbazine). After 6 cycles, he remained asymptomatic and symptoms of digital ischemia were completely resolved. It was concluded that the presence of acral vascular syndromes should alert the physician about the possibility of underlying malignant disease. Prompt investigation and treatment should be rapidly performed to avoid digital sequelae.

## 1. Introduction

Digital ischemia is associated with atherosclerotic, thromboembolic, or connective tissue diseases. Less often, it can be related to malignancy. It can precede, coincide, or follow the diagnosis of cancer; in contrast to this, the majority of patients develop digital ischemia and cancer simultaneously. Paraneoplastic vascular acrosyndromes (Raynaud's syndrome, acrocianosis, and acronecrosis) are associated more frequently with adenocarcinoma, in particular breast, lung, and gastrointestinal carcinoma, and less frequently with hematological malignancies [1, 2]. The best described pathogenic mechanisms so far are cryoglobulinemia or cold agglutinin disease in acral syndrome related to lymphoma, but there are many other unknown events in those who lack these factors [1]. We present a rare case of a patient with digital ischemia associated with classical Hodgkin's lymphoma.

## 2. Case Report

**Figure 1:**
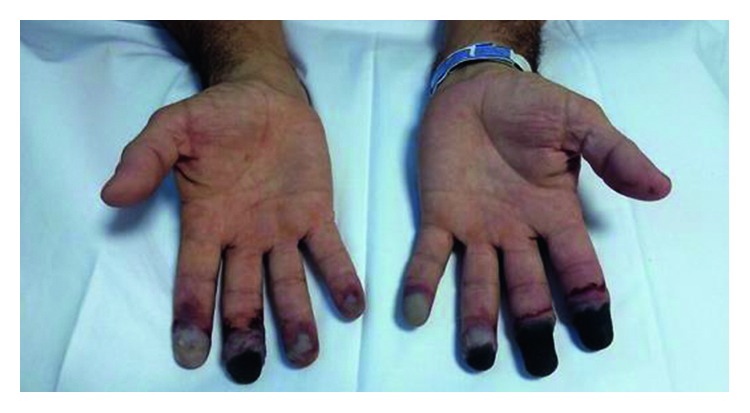
Necrotic lesions on the distal phalanges.

A 45-year-old male, smoker, who presented a 10-day history of painful, cyanosis, and progressive digital necrosis in both hands. In the four previous months, he noticed painless mass in the right axillary gap, drenching night sweats, and 5 kg weight loss. No clinical features of connective tissue disease were acknowledged such as systemic lupus erythematosus or systemic sclerosis (e.g., photosensitivity, arthralgias, arthritis, skin hardening, and sicca syndrome). Physical examination at admission highlighted necrotic lesions on the distal phalanges of both hands (except the thumbs) (Figure 1), enlarged lymph nodes in right axillary (3 cm), and right supraclavicular gaps (4 cm). All pulses were palpable. A computed tomographic scan evidenced right axillary adenopathic conglomerate (6 cm), right supraclavicular gap (5 cm), and multiple retroperitoneal adenopathies (<10 cm). Initial investigation showed hemoglobin 113 g/L, hematocrit 42%, total white cell 18.3 × 10^9^/L, lymphocyte count 4.8 × 10^9^/L, platelets 280,000/mm^3^, and albumin 2.4 g/dl. Electrolytes, liver function, lipidic profile, and serum protein electrophoresis (SPEP) were normal. Results of coagulation test were also normal. Serum complement levels and serologic test for rheumatoid factor, antinuclear, antineutrophil-cytoplasmatic, lupus anticoagulant, anticardiolipin, anti-B2 glycoprotein 1, cryoglobulins, and cryoaglutinins were negative. Hereditary thrombophilia was not evaluated. Human immunodeficiency virus and hepatitis B and C viruses were negative. Arteriography of upper limbs demonstrated a distal stop in all bilateral digital arteries. Transthoracic echocardiogram was normal. He was initially treated for 10 days with anticoagulation with low-molecular-weight heparin, antiplatelet agents such as acetylsalicylic acid at 100 mg once daily and nifedipine, without improvement of ischemia.

**Figure 2:**
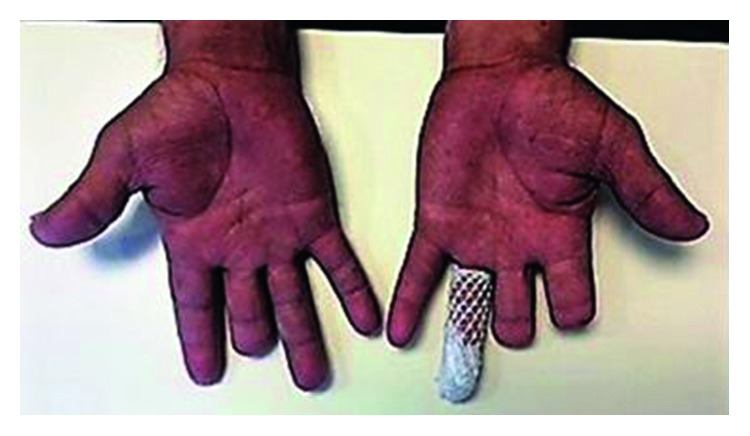
Resolution of necrotic lesions after 3 cycles of ABVD protocol (3 months from the HL diagnosis). Amputation of three phalanges was required.

**Figure 3:**
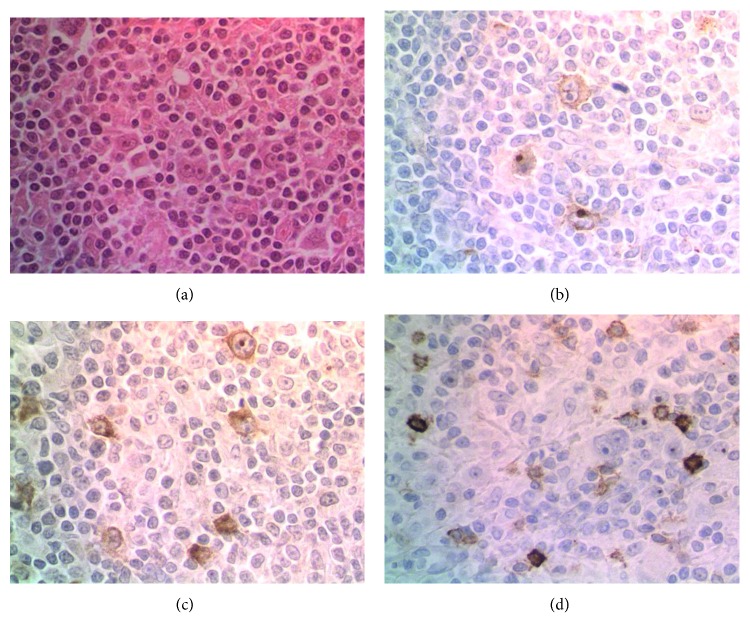
Lymph node biopsy sample. (a) Typical morphology of Reed–Sternberg cells, surrounded by a background of reactive inflammation, nodular sclerosis pattern (HE stain ×40). Immunohistochemistry staining of the Reed–Sternberg population showed expression of (b) CD15 and (c) CD30. (d) CD20 expression was negative.

As other common etiologies had been ruled out with investigations, digital ischemia was interpreted as a paraneoplastic phenomenon. Corticosteroids (hydrocortisone 200 mg every 8 hours) and a bolus of 1 g intravenous cyclophosphamide were indicated. A significant decline of pain, cyanosis, and progression of ischemic lesions was noticed after 6 days of treatment. However, amputation of three phalanges was required due to necrosis. After surgery, a delay for healing due to acrosyndrome was not observed (Figure 2). The use of iloprost was not discussed due to the evolution. Biopsy of axillary nodes demonstrated nodular sclerosis classical Hodgkin's lymphoma (HL) (Figure 3).

Bone marrow was not infiltrated. Classical HL stage IIIb was confirmed. The patient then started conventional ABVD protocol (doxorubicin, bleomycin, vinblastine, and dacarbazine), which was well tolerated. After 6 cycles, he remained asymptomatic, and symptoms of digital ischemia were completely resolved. At the end of treatment, positron emission tomography/computed tomography was negative, confirming complete remission of HL. Follow-up was performed monthly during 6 months and then every 3 months. After 18 months, the patient remains in remission of HL, with no evidence of digital ischemia recurrence.

## 3. Discussion

Acral vascular syndromes may be encountered in various clinical conditions, such as connective tissue diseases, thromboembolic diseases, or atherosclerotic diseases and more rarely in malignancies [1, 2]. Among malignant disorders, solid tumors are the most frequently reported, mainly adenocarcinomas (lung, breast, and digestive), followed by hematooncological diseases, predominantly lymphoproliferative disorders [1–3]. Acral vascular syndromes can occur concomitantly, precede or be diagnosed after cancer [2]. No differences in the clinical presentation of these syndromes have been mentioned depending on the underlying etiology. Hodgkin's lymphoma may associate paraneoplastic phenomena [4]. Digital ischemia as a paraneoplastic syndrome of Hodgkin's disease is an unusual finding [4, 5]. There are very little data in the literature that was reviewed, mostly case reports, as shown in Table 1 [5–7]. In fact, pathogenic mechanism of paraneoplastic acral vascular syndromes is poorly understood and likely multifactorial. Vasoconstrictive substances produced by the tumor cells, thromboembolic mechanism with embolization by microfragments of the tumor directly in the blood, circulating procoagulant factors, and impaired anticoagulant and fibrinolitic pathways are involved. Hyperstimulation of the sympathetic nervous system as a result of compression of the cervical plexus by the tumor and cryoglobulins of some cancer patients support the hypothesis of an immunologic mechanism [1, 2, 8, 9]. Outcomes of malignancy and digital ischemia may not be parallel. However, in the majority of cases, treatment of the tumor resolved vascular involvement [2].

This case illustrates Hodgkin's lymphoma presenting with acral necrosis which required digital amputations. Laboratory studies such as a peripheral hemogram, lipidic profile, serologic test (serum complements levels, rheumatoid factor, antinuclear, antineutrophil-cytoplasmatic, lupus anticoagulant, anticardiolipin, and anti-B2 glycoprotein 1), SPEP, cryoglobulin, and cold agglutinin and imaging techniques such as echocardiography should be used for initial assessment of ischemia. Arteriography is useful to evaluate the extent of compromise. Clinical presentation with enlarged adenopathies and constitutional symptoms in this case proved to be related to lymphoma. The treatment of the tumor solved the reversible vascular affectation suggesting a paraneoplastic mechanism. In addition, the patient has another risk factor for the development of digital ischemia such as smoking. This condition in fact could have induced a digital arteritis, and furthermore, the lymphoma could have been the trigger of necrosis in this context.

In conclusion, the presence of acral vascular syndromes should alert the physician about the possibility of an underlying malignant disease. In most cases, the treatment of the malignancy would induce to the resolution of the vascular involvement. Prompt investigation and treatment should be rapidly performed to avoid digital sequelae.

## Figures and Tables

**Table 1:** Summary of case reports associating digital ischemia with Hodgkin's lymphoma.

Date	Author	Sex	Age	Histological subtype	Symptoms	Treatment	Course
1994	Halpern et al. [5]	Male	52	Nodular lymphocyte predominant	Digital ischemia and gangrene	Cervical sympathectomy; mustine, vinblastine, procarbazine, and prednisolone (6 cycles)	Complete remission and resolution of digital ischemia

2011	Poiraud et al. [6]	Male	59	Classic	Digital necrosis, weight loss, and adenopathies	Vinblastine alone (2 cycles) (anthracyclines and bleomycin contraindicated)	Died suddenly at home at 3 months of diagnosis

2006	Solak et al. [7]	Male	17	Classic, nodular sclerosis	Acrocyanosis, fever, and adenopathies	Doxorubicin, bleomycin, vinblastine, and dacarbazine (6 cycles)	Complete remission and resolution of acrocyanosis
